# Stabilization of *Candida antarctica* Lipase B (CALB) Immobilized on Octyl Agarose by Treatment with Polyethyleneimine (PEI)

**DOI:** 10.3390/molecules21060751

**Published:** 2016-06-08

**Authors:** Sara Peirce, Veymar G. Tacias-Pascacio, Maria Elena Russo, Antonio Marzocchella, José J. Virgen-Ortíz, Roberto Fernandez-Lafuente

**Affiliations:** 1 Departamento de Biocatálisis, Instituto de Catálisis-CSIC, C/Marie Curie 2, Campus UAM-CSIC Cantoblanco, 28049 Madrid, Spain; sara.peirce@unina.it (S.P.); vey_pascacio@live.com (V.G.T.-P.); 2 Dipartimento di Ingegneria Chimica, dei Materiali e della Produzione Industriale, Universita’ degli Studi di Napoli Federico II, 80125 Napoli, Italy; marzocch@unina.it; 3 Unidad de Investigación y Desarrollo en Alimentos, Instituto Tecnológico de Veracruz, Calzada Miguel A. de Quevedo 2779, 91897 Veracruz, Mexico; 4 Istituto di Ricerche sulla Combustione—Consiglio Nazionale delle Ricerche, 80125 Napoli, Italy; m.russo@irc.cnr.it

**Keywords:** reversible immobilization, interfacial adsorption, PEI modification, enzyme stabilization, enzyme physical intermolecular crosslinking

## Abstract

Lipase B from *Candida antarctica* (CALB) was immobilized on octyl agarose (OC) and physically modified with polyethyleneimine (PEI) in order to confer a strong ion exchange character to the enzyme and thus enable the immobilization of other enzymes on its surface. The enzyme activity was fully maintained during the coating and the thermal stability was marginally improved. The enzyme release from the support by incubation in the non-ionic detergent Triton X-100 was more difficult after the PEI-coating, suggesting that some intermolecular physical crosslinking had occurred, making this desorption more difficult. Thermal stability was marginally improved, but the stability of the OCCALB-PEI was significantly better than that of OCCALB during inactivation in mixtures of aqueous buffer and organic cosolvents. SDS-PAGE analysis of the inactivated biocatalyst showed the OCCALB released some enzyme to the medium during inactivation, and this was partially prevented by coating with PEI. This effect was obtained without preventing the possibility of reuse of the support by incubation in 2% ionic detergents. That way, this modified CALB not only has a strong anion exchange nature, while maintaining the activity, but it also shows improved stability under diverse reaction conditions without affecting the reversibility of the immobilization.

## 1. Introduction

Lipases are the most used enzymes in biocatalysis due to their good activity, specificity, selectivity and robustness in a variety of reaction media [1,2,3]. They have a peculiar catalytic mechanism based in deep conformational changes called interfacial activation that permits them to act in the hydrolysis of insoluble drops of oils [4,5], and this causes them to have a tendency to become adsorbed on any hydrophobic surface [6,7,8,9]. In order to use them as industrial biocatalysts, immobilization is required in many instances in order to facilitate their reuse [10,11,12,13]. This step should be used to improve other enzyme features. Usually the focus is on enzyme stability, but other enzyme properties may be also improved, such as purity, activity, selectivity or specificity [14,15,16,17,18].

An ideal industrial enzyme immobilization method must allow one to improve all enzyme features in a very simple way [15]. In this sense, immobilization of lipases on hydrophobic supports [6,7] may be noted as an useful tool to improve lipase properties: this immobilization strategy permits the specific lipase immobilization (purification) [6], involves the surroundings of the active center of the lipase and, in most cases, the open form of the lipase (hyperactivation and stabilization) [19,20], may be used to tune enzyme properties [21], and is very simple to perform and reversible, enabling support reuse [6,7]. Unfortunately, it has a drawback: the enzyme may be released to the medium when exposed to high temperatures or high concentrations of organic cosolvents during operation [22].

To solve this problem, some authors have chemically crosslinked the lipases using aldehyde dextran [23,24] or glutaraldehyde [25]. The intermolecular crosslinking causes the enzymes to be only desorbed when all the enzymes in the aggregate simultaneously break their interaction with the support. The system works, but converts a reversible method into an irreversible one, which makes both enzyme and support disposable after enzyme inactivation.

Another explored solution has been the use of heterofunctional supports having acyl groups (to permit lipase immobilization via interfacial activation) and some moieties able to form covalent attachments [22,26,27,28,29,30,31,32]. This treatment prevents enzyme desorption and improves enzyme stability, even enabling the use of the enzyme under conditions where a support with only acyl groups cannot maintain the enzyme immobilized. The method also permits a partial reactivation of some enzymes [33], but again transforms a reversible method in an irreversible one. In a further step, heterofunctional acyl-ionic supports have been designed [34,35]. However, the results were quite complex, although enzyme release was reduced and the reversibility of the immobilization was maintained, the immobilized lipase was destabilized in some cases [34,35].

Polyethylenimine has been used in biocatalyst design with different objectives: generation of hydrophilic environments to reduce the concentration of solvents molecules or oxygen, stabilization of multimeric enzymes, to keep the activated form of lipases, to prevent oxidation, *etc.* [36,37,38,39,40,41,42,43,44,45,46]. The open structure of the coating does not produce steric hindrance to the entry of small substrates, and it may just produce some partition of hydrophobic substrates from the enzyme environment. In some instances the enzyme activity is even increased after coating [47]. As the interaction is reversible, the use of ionic detergents, guanidine, *etc.* may permit the release of the enzyme and the PEI, permitting the reuse of the support. However, to date, it has been never analyzed if the positive effects of the enzyme coating after enzyme immobilization may be, at least in part, due to the intermolecular physical crosslinking between different immobilized enzyme molecules, avoiding that way the enzyme desorption from the support.

As model enzyme we have utilized the most widely used lipase, lipase B from *Candida antarctica* (CALB), a robust enzyme with many applications [48,49]. It has a very small lid that does not fully seclude the active center from the medium [49], but has been successfully immobilized on a handful of hydrophobic supports [22,25]. Moreover, the PEI coating of the immobilized CALB has permitted to design an immobilized CALB form with good properties to be used as “immobilization support” to co-immobilize other enzymes on the already immobilized lipase via anion exchange. The results of this protocol to co-immobilize lipase and other enzymes have been recently published but the effects of the modification on the enzyme properties have not been properly analyzed [50]. Furthermore, there is no knowledge whether this treatment does or does not produce some enzyme crosslinking. Thus, the objective of this paper was to characterize the optimal PEL-OCCALB preparation useful for enzyme co-immobilization. To reach this goal, a large excess of PEI was used trying to prevent that each PEI molecule may cover a large area of enzyme molecule, because a large polymeric bed is intended. This protocol should produce a large shell of ion polymer coating the enzyme surface, and may maximize the partition effects of this coating in the presence of hydrophobic molecules, like organic solvents. Moreover, we have paid special attention to the possibility of achieve some physical intermolecular crosslinking that could explain, at least partially, some of the positive effect.

## 2. Results and Discussion

### 2.1. Immobilization of CALB on Octyl-Agarose

**Figure 1:**
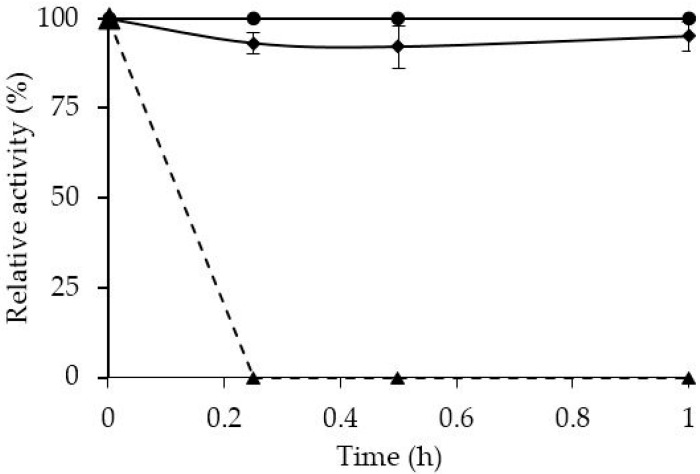
Immobilization courses of CALB at pH 7 on octyl agarose at low ionic strength. Rhombi, solid line: suspension; triangles, dashed line: supernatant; circles, solid line: reference.

Figure 1 shows that CALB is rapidly (30 min) and fully immobilized on OC with low effect on enzyme activity. The lack of a real lid that isolates the enzyme active center [51] caused the adsorption on octyl agarose to fail to produce the usual increase on enzyme activity found with other lipases [6].

### 2.2. Effects of PEI Modification on Enzyme Activity/Stability

The coating of the enzyme with PEI did not produce any effect on enzyme activity. This result was positive as the presence of the shell covering the enzyme surface could produce some problems to the entry of the substrate. However the open structure of the polymer has not effect on enzyme activity, as it has been found with many other enzymes other than CALB [37,39,45].

**Figure 2:**
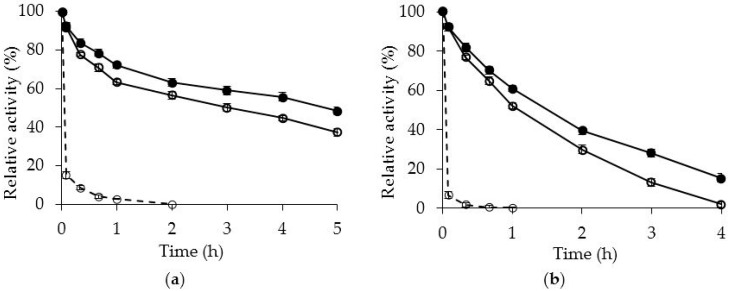

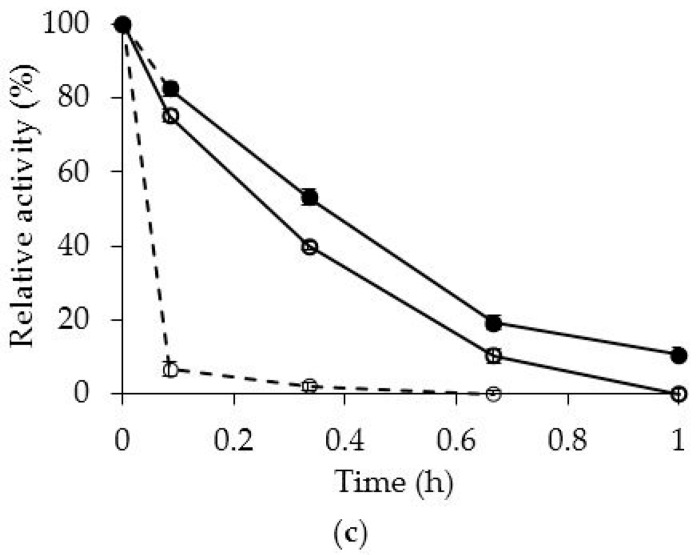
Thermal inactivation courses of the OCCALB and OCCALB-PEI biocatalysts. Panel (**a**): 80 °C and pH 5; panel (**b**): 70 °C and pH 7; panel (**c**): 60 °C and pH 9. Open circles, solid line: OCCALB; closed circles, solid line: OCCALB PEI; dashed line: free CALB.

Figure 2 shows that while immobilization on OC produced a significant stabilization, the PEI treatment of the immobilized enzyme has a marginal effect on enzyme stability, with small increases on enzyme stability at the three studied pH values. It should be considered that the PEI may be introducing intra- and inter-molecular crosslinkings, but the weak nature of the bonds and the flexible nature of the polymer did not produce a significant change in enzyme stability [52,53].

The results are much better than when using amino-octyl supports [35] to improve the enzyme immobilization, because that support produced enzyme destabilization at pH 5 and 7 and only some stabilization was observed at pH 9. Using glutamic-octyl [34] the stability decreased under acid media value while it increased more clearly than using PEI at neutral pH value. Glutaraldehyde crosslinking gave better results in terms of stabilization [25], because the small size of the crosslinking reagent permitted a higher enzyme rigidification, the combination of inter and intramolecular crosslinking produced a significant stabilization. However, this treatment avoided the enzyme desorption and therefore, transformed this reversible immobilization into an irreversible one, precisely want we wanted to prevent.

**Figure 3:**
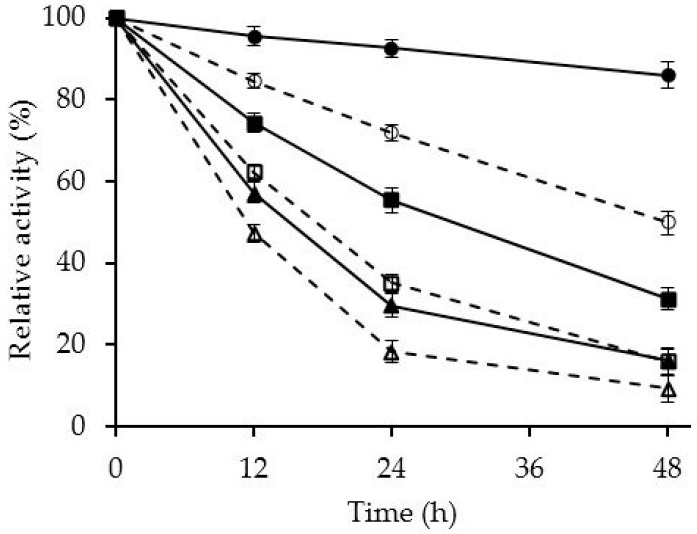
Effect on enzyme activity of the incubation of the immobilized CALB in the presence of different organic solvents. Enzyme preparations were incubated in mixtures of organic solvents/100 mM Tris-HCl pH 7 at 40 °C. Solid line: OCCALB-PEI biocatalyst; dashed line: OCCALB biocatalyst; circles: 80% 1,4-dioxane; squares: 45% acetonitrile; triangles: 70% ethanol.

Figure 3 shows that this physical modification of the enzyme with PEI has a more significant effect on the stability of the enzyme in the presence of different organic cosolvents. Although OCCALB is already quite stable in this medium, the coating with PEI significantly improved the stability, mainly in the presence of hydrophobic dioxane.

This stabilization produced by PEI coating in organic solvents has been usually associated to the partition of the hydrophobic organic solvents, but the possibility of enzyme intermolecular physical crosslinking has not been analyzed. The use of amino-octyl [35] almost did not alter the enzyme stability in the presence of organic cosolvents, while glutamic-octyl [34] permitted stabilizations of even 10 folds in these media, higher than those described here. Glutaraldehyde, as in thermal inactivations, provided a higher stabilization (more than 10-fold) than the coating with PEI [25].

### 2.3. Effect of PEI Treatment in the Desorption of OCCALB by Incubation in non Ionic Detergents

**Figure 4:**
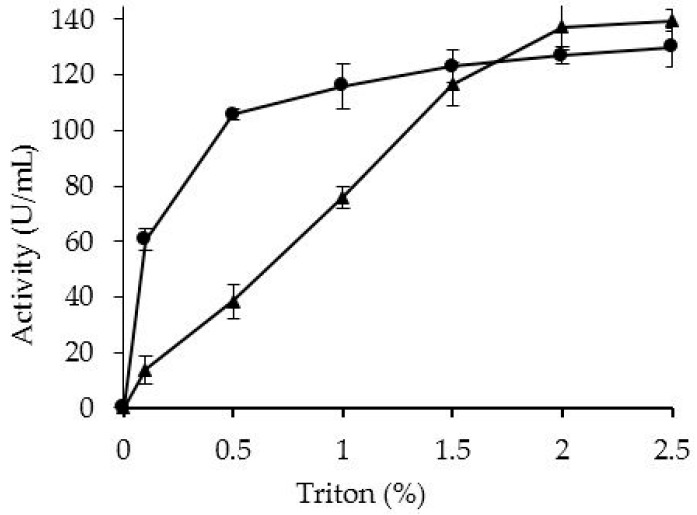
Desorption of the CALB from the OCCALB (circles) and OCCALB-PEI (triangles) derivatives with Triton X-100.

OCCALB and OCCALB-PEI were incubated in growing concentrations of Triton X-100 (Figure 4). The PEI treatment increased the amount of detergent required to desorb CALB from the octyl support. For example using 0.5% almost all activity was released from OCCALB and only a 33% was released from OCCALC-PEI. This higher difficulty of CALB release after PEI treatment suggested that some CALB molecules could be physically crosslinked and that could hinder enzyme desorption. However, finally most enzyme molecules could be desorbed, suggesting that the physical crosslinking using this protocol is not maximized.

### 2.4. Effect of PEI Modification in the Release of CALB Molecules to the Medium during Inactivation

**Figure 5:**
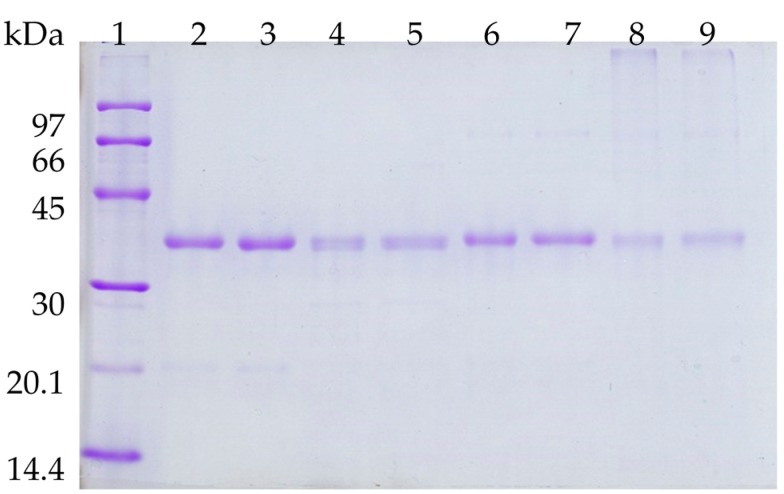
SDS-PAGE analysis of OCCALB and OCCALB-PEI derivatives under different treatments. Lane 1: low molecular weight protein standard from GE Healthcare. Lane 2: OCCALB; lane 3: OCCALB-PEI; lane 4: OCCALB and lane 5: OCCALB-PEI incubated at pH 5 and 80 °C. Lane 6: OCCALB and lane 7: OCCALB-PEI incubated at pH 7 and 70 °C. Lane 8: OCCALB and lane 9: OCCALB-PEI incubated at pH 9 and 60 °C.

**Figure 6:**
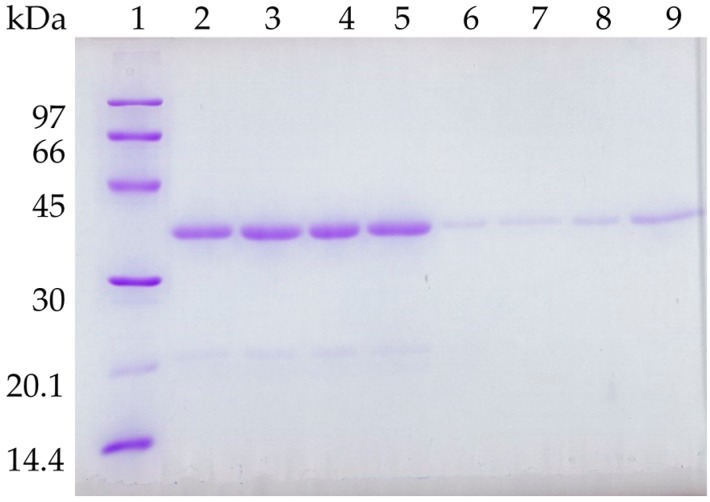
SDS-PAGE analysis of OCCALB and OCCALB-PEI derivatives with organic solvents. Lane 1: low molecular weight protein standard from GE Healthcare. Lane 2: OCCALB; lane 3: OCCALB-PEI; lane 4: OCCALB and lane 5: OCCALB-PEI incubated in 90% dioxane; lane 6: OCCALB and lane 7: OCCALB-PEI incubated in 70% ethanol; lane 8: OCCALB and lane 9: OCCALB-PEI incubated in 45% acetonitrile.

It has been described that lipase molecules are desorbed to the medium from OC supports during solvent and thermal inactivations [22]. To check this, the biocatalysts submitted to inactivation were recovered and the amount of protein still attached to the support was analyzed by SDS-PAGE. Figure 5 shows that the OCCALB desorbs part of the enzyme (the intensity of the protein band is lower in the inactivated biocatalysts following densitometry analyses of the gels) during thermal inactivation, mainly at pH 9. The treatment with PEI reduced this decrease in the CALB band (around 50% more protein could be observed). Figure 6 shows that most of the enzyme remained immobilized when incubated in dioxane for 48 h, while ethanol and acetonitrile was able to desorb higher amounts of enzyme. PEI reduced this enzyme release caused by the solvent, mainly using acetonitrile (threefold more protein remained in the biocatalysts after PEI treatment).

### 2.5. Reuse of the Support

The washing of the OCCALB-PEI with 2% CTAB or SDS at 40 °C permitted to fully eliminate the CALB adsorbed on the support, even after enzyme inactivation (it has been reported that the inactivated enzyme may become strongly adsorbed on the support) [54]. The support, after washing with water, could be reused for five cycles while maintaining the immobilization rate, enzyme stability and activity.

## 3. Materials and Methods

### 3.1. Materials

Solution of lipase B from *Candida antarctica* (CALB) (6.9 mg of protein/mL) was a kind gift from Novozymes (Alcobendas, Spain). Polyethylenimine (PEI) (MW 25,000), Triton X-100 and *p*-nitrophenyl butyrate (*p*-NPB) were purchased from Sigma-Aldrich (St. Louis, MI, USA). Octyl Sepharose CL-4B beads was from GE Healthcare Bio-Sciences (Uppsala, Sweden). Electrophoresis purity reagents were obtained from Bio-Rad (Hercules, CA, USA). All other reagents were of analytical grade. Protein concentration was estimated by the Bradford dye binding method at 595 nm [55] using bovine serum albumin as a standard.

### 3.2. Standard Measure of Enzyme Activity

The enzyme activity assay was performed by measuring the increase in the absorbance at 348 nm (isobestic point) produced by the released *p*-nitrophenol in the hydrolysis of 0.4 mM *p*NPB in 25 mM sodium phosphate at pH 7 and 25 °C (ε under these conditions is 5150 M^−1^·cm^−1^). A spectrophotometer with a thermostated cell and with continuous magnetic was used. To initiate the reaction, 50–100 µL of lipase solution or suspension were added to 2.5 mL of substrate solution. One international unit of *p*NPB activity was defined as the amount of enzyme necessary to hydrolyze 1 µmol of *p*NPB min^−1^ (U) under the conditions described above.

### 3.3. Immobilization of CALB on Octyl (OC) Supports

The standard immobilization was performed using 2 mg of lipase per g of wet support (20 units/g). CALB solution was diluted in the corresponding volume of 5 mM sodium phosphate at pH 7. Then, OC support was added [6]. The activity of both supernatant and suspension was followed using *p*NPB assay. After immobilization the suspension was filtered and the immobilized biocatalyst enzyme was exhaustively washed with distilled water.

### 3.4. Modification of OCCALB with PEI

A 50 mL solution of 10% PEI (*w*/*v*) was prepared in 5 mM sodium phosphate and the pH was adjusted at pH 7. Then, 5 g of OCCALB was suspended and submitted to gentle stirring for 2 h. Afterwards, the modified enzyme was washed with an excess of distilled water to eliminate the free PEI. The biocatalyst was stored at 4 °C in wet conditions.

### 3.5. Thermal Inactivation of OCCALB and OCCALB-PEI Preparations

Immobilized biocatalyst (1 g) was suspended in 25 mM sodium acetate (5 mL) at pH 5, sodium phosphate at pH 7 or sodium bicarbonate buffer at pH 9 at different temperatures. Periodically, samples were withdrawn and the activity was measured using *p*NPB.

### 3.6. Inactivation of OCCALB and OCCALB-PEI in the Presence of Organic Co-Solvents

The biocatalysts were incubated in mixtures of ethanol, acetonitrile or 1,4-dioxane/100 mM Tris-HCl buffer (pH 7) at different temperatures. Periodically, samples were withdrawn and the activity was measured using *p*NPB as described above.

### 3.7. Desorption of the CALB from the Supports

Samples of 1 g of the immobilized biocatalysts were suspended in 10 mL of 5 mM sodium phosphate buffer at pH 7. Then, Triton X-100 was progressively added to a final concentration of 2.5% (*v*/*v*). Intervals of 30 min were allowed before taking a sample of the supernatant to determine the released enzyme and performing a new detergent addition [22]. Finally, 100% of the enzyme contained in the support was released from the support.

### 3.8. SDS-PAGE Experiments

SDS-polyacrylamide gel electrophoresis experiments were performed according to Laemmli [56] using a Miniprotean tetra-cell (Bio-Rad), 14% running gel in a separation zone of 9 cm × 6 cm, and a concentration zone of 5% polyacrylamide. One hundred milligrams of the immobilized enzyme samples was re-suspended in 1 mL of rupture buffer (2% SDS and 10% mercaptoethanol), boiled for 8 min and a 10 μL aliquot of the supernatant was used in the experiments. This treatment released all enzyme just interfacially activated on the support [6]. Gels were stained with Coomassie brilliant blue. A low molecular weight calibration kit for SDS electrophoresis (GE Healthcare) was used as a molecular weight marker (14.4–97 kDa).

### 3.9. Reuse of the Support

These experiments were performed in syringes with a silica plate in the outlet to prevent loss of support. 1 g of support was loaded with CALB, treated with PEI, and incubated in 2% CTAB or SDS at different temperatures (two washings of three volumes of detergent solution for 5 min). Then, the support was washed 10 times with 10 mL of distilled water to eliminate the detergent (protocols not optimized).

## 4. Conclusions

The coating of octyl-CALB with PEI following the protocol successfully applied to enzyme co-immobilization has no negative effects on enzyme activity and has positive effects on enzyme stability, mainly in mixtures of organic cosolvents/aqueous buffers, even if this protocol was not optimized for this purpose. The physical coating of the enzyme molecules with PEI reduces enzyme release from the support, both in incubation in nonionic detergents or after enzyme inactivation (thermal or organic solvent) suggesting that some intermolecular physical crosslinking may be relevant for the stabilization results. However, it has been previously described that this modification may also reduce the concentration of solvent in the enzyme environment (e.g., the highest stabilization is in the presence of dioxane, where OCCALB did not release almost enzyme molecules to the medium), therefore the stabilization may be caused by several factors, but the physical intermolecular crosslinking can also play some role. This strategy for enzyme stabilization did not avoid the possibility of desorbing the enzyme and reusing the support after enzyme inactivation because all is reversible: enzyme adsorption on this support is via hydrophobic interaction; PEI crosslinking is via ion exchange. It should be considered that this “physical” enzyme intermolecular crosslinking has been observed using a protocol designed to get a further enzyme adsorption on the PEI coated CALB and not to stabilize the enzyme. Thus, the further optimization of the PEI enzyme coating may produce a better PEI intermolecular physical crosslinking (e.g., chemical succinylation of the enzyme surface, PEI size and concentration). This preliminary results suggest that the strategy may be of general usefulness to prevent any enzyme desorption from supports where the immobilization is not irreversible. Compared to the use of ion-octyl supports, the results on enzyme thermostability were clearly better using this protocol of PEI coating, the results in stability in organic cosolvents/aqueous medium was under that obtained using glutamic, but considering that the optimization was performed to get a good conimmobilization of a second enzyme, the results are quite promising. A proper coating with ionic polymers of CALB immobilized on octyl supports (therefore with the enzyme molecules packed together) should permit to further improve the results.

## Abbreviations

The following abbreviations are used in this paper:

CALB	*Candida antarctica* lipase B
OC	octyl-Sepharose
OCCALB	CALB immobilized on OC
PEI	polyethyleneimine
*p*npb	*p*-nitrophenyl butyrate
